# Soybean oil addition to wheat bran-based diet improves laying hens’ performance

**DOI:** 10.14202/vetworld.2023.1572-1575

**Published:** 2023-07-31

**Authors:** Mariana Novela, Sónia Carlitos Pinto, Angélica Tembe, Emmanuel Paulo, Marcos Mabasso, Albino Gove, Abilio Paulo Changule, Leonel António Joaquim, Ramos Tseu, Filomena dos Anjos, Custódio Gabriel Bila

**Affiliations:** 1 Section of Animal Pathology, Department of Animal and Public Health, Faculty of Veterinary Medicine, Eduardo Mondlane University, Maputo, Mozambique; 2 Department of Basic Sciences, Faculty of Veterinary Medicine, Eduardo Mondlane University, Maputo, Mozambique; 3 Directorate of Animal Science, Agricultural Research Institute of Mozambique, Maputo, Mozambique; 4 Angónia Reseach Station, Agricultural Research Institute of Mozambique, Maputo, Mozambique; 5 Section of Animal Nutrition, Faculty of Veterinary Medicine, Eduardo Mondlane University, Maputo, Mozambique

**Keywords:** hens, laying, soybean oil, wheat bran

## Abstract

**Background and Aim:**

Wheat bran (WB) is used extensively in animal feed. Despite its nutritional value, its use is limited because of its high-fiber content. We evaluated the effect of soybean oil on laying hen performance with maize meal partly replaced by WB.

**Materials and Methods:**

Thirty-six ISA Brown laying hens, 40 weeks old, were used in a completely randomized design in which laying hens were distributed in individual cages, with three replications of four birds and assigned to three treatments: T1 (basal diet), T2 (60% basal diet + 20% maize meal + 20% WB), and T3 (60% basal diet + 20% maize meal + 17.5% WB + 2.5% soybean oil).

**Results:**

Compared with the control group (T1), replacing 20% of yellow maize with WB (T2) did not affect average live weight, egg laying rate, soft-shelled egg production, egg mass, feed conversion per dozen eggs, or laying hen viability (p > 0.05). When 20% of the maize meal was replaced with WB, feed intake and feed conversion per egg mass increased (p < 0.05). Furthermore, adding 2.5% soybean oil to feed containing WB improved laying hen performance by significantly reducing feed conversion per egg mass (p < 0.05).

**Conclusion:**

Adding 2.5% soybean oil to diets containing WB instead of 17.5% yellow maize improved the feed conversion per egg mass performance of laying hens.

## Introduction

Poultry production is the largest and fastest-growing agricultural subsector, especially in developing countries [1, 2]. Finding alternatives to conventional poultry feed sources is becoming increasingly important in some countries to meet rising demand [2, 3]. Wheat bran (WB), a raw material rich in dietary fiber, has gained considerable interest because of its beneficial effect on gut physiology and the increasing global demand for animal-based protein sources. Wheat bran contains a lot of dietary fibers, protein, starch, minerals, and bioactive compounds [4]. As a byproduct of the milling industry, up to 150 million tons of WB are produced globally yearly [4].

Evidence of increasing crop competition between humans and animals has prompted research into using agricultural by-products such as WB in animal feed [5, 6]. Several studies [7–11] have investigated the use of WB in laying hen feed formulations. Across studies, increased feed intake and poorer feed conversion have been reported as common findings. One reason for the described poor performance could be the lower energy content of WB. To the best of our knowledge, little information exists on the combined use of WB and high-energy ingredients in laying hen diets [9, 12]. We hypothesized that incorporating high-energy ingredients like soybean oil would improve laying hen performance when replacing a maize meal with WB [13].

Soybean oil is a high-quality consumable and energetic vegetable oil for humans, derived from a high-quality bean used in animal feed worldwide. Soybean oil has a high-energy level because of its high percentage of (poly) unsaturated fatty acids, which are well absorbed and used as a source of energy by the animal. It contains 24% monounsaturated fatty acids and 61% polyunsaturated fatty acids and is high in sterols and Vitamins E and K [11].

This study aimed to determine the effect of soybean oil addition on the performance of laying hens in diets where the yellow maize meal was partly replaced with WB.

## Materials and Methods

### Ethical approval

The study was approved by Scientific Board of the Faculty of Veterinary Medicine, Eduardo Mondlane University (Number: 895FAVET).

### Study period and location

This study was conducted from April to August 2022 at the animal farm of the Faculty of Veterinary Medicine, Eduardo Mondlane University, Maputo, Mozambique.

### Experimental design

For the 8-week experiment, 36 Institute de Seléction Animale (ISA) Brown laying hens that were

laying hens had been producing eggs for 30 weeks before the start of the experiment. The experiment was performed in a battery cage system housed in a deep pit with natural ventilation and window illumination from both artificial and natural sources.

In a completely randomized design, laying hens were divided into three groups of 12 laying hens, each with three replications of four birds, and assigned to one of three treatments: T1 (basal diet), T2 (60% basal diet + 20% maize meal + 20% wheat bran), and T3 (60% basal diet + 20% maize meal + 17.5% wheat bran + 2.5% soybean oil). Table-1 shows the composition of the experimental diets and their calculated nutrient levels. Wheat bran and soybeans were purchased from local industry market.

**Table-1:** Composition of experimental diets.

Ingredients and calculated composition	Diets (%)		

	1	2	3
Yellow maize	60	40	40
Soya bean meal	36.65	36.65	36.65
Wheat bran	0	20	17.5
Soya bean oil	0	0	2.5
Trace mineral premi×^1^	3	3	3
Vitamin premi×^2^	0.1	0.1	0.1
Dicalcium phosphate	0.25	0.25	0.25
Total	100	100	100
Calculated composition			
Megajoule (MJ )	13.80	12.87	13.60
Protein	14.5	17.62	17.1
Fiber	7	8.06	7.86
Ether extract	2.5	3.1	5.522
Lysine	0.75	0.75	0.731
Methionine	0.25	3.1	5.52
Calcium	4	4.04	3.947
Total phosphorus	0.4	0.702	0.684

^1^Trace mineral premix provided the following per kilogram of diet: Mn, 80 mg; Fe, 60 mg; Zn, 60 mg; Cu, 5 mg; Co, 0.2 mg; I, 1 mg; Se, 0.15 mg; Ca, 446.9 mg. ^2^Vitamin premix provided the following per kilogram of diet: Vitamin A 12,000 IU; cholecalciferol, 2000 IU; Vitamin E, 35 IU; Vitamin K_3_, 5 mg; thiamin, 3 mg; riboflavin, 6 mg; niacin, 20 mg; Ca-d-pantothenate, 6 mg; pyridoxine, 5 mg; Vitamin B_12_, 15 µg, folacin, 0.75 mg, D-biotin, 45 µg, choline chloride, 125 mg; Vitamin C, 50 g

The birds were subjected to the same routine management conditions. Water was consumed ad-libitum, while the feed was given at 120 g per bird per day; the leftovers were weighed, and daily feed intake was calculated. Throughout the experiment, all groups received identical treatment.

The initial body weights and egg yield ratios of all experimental laying hens were recorded at the start of the experiment and then weekly. Daily egg yields, soft eggshell yields, egg weights, and mortality were recorded, and weekly feed consumption was recorded. The feed intake, egg yield ratio, soft eggshell yield ratio, feed conversion per egg mass, feed conversion per dozen eggs, and mortality ratios were calculated every week.

### Statistical analysis

The generated data were analyzed with a one-way analysis of variance using SPSS software v 25 (IBM Corp., NY, USA. The Turkey test was used to compare treatment means at a 5% significance level.

## Results

Table-2 displays the performance results of laying hens from the three treatments. Compared to the control group (T1), replacing 20% of yellow maize with WB (T2) did not affect average live weight, egg laying rate, soft-shelled egg production, egg mass, feed conversion per dozen eggs, or laying hen viability (p > 0.05). When 20% of the maize meal was replaced with WB, feed intake and feed conversion per egg mass increased (p < 0.05). Furthermore, adding 2.5% soybean oil to WB-containing feed improved laying hen feed conversion per egg mass.

**Table-2:** Effects of partial replacement of yellow corn with wheat bran and soya bean oil.

Parameters	T1	T2	T3	CV%
Laying hens average weight (g)	1851	1729	1784	12.40
Feed intake (kg)	6.175	6.539^a^	6.2124^b^	0.60
Egg production ratio (%)	94.933	93.793	92.563	4.80
Soft eggshell ratio (%)	0	0.0483	0.0997	243.55
Egg mass (kg)	1.7017	1.7147	1.6930	3.97
Feed conversion per egg mass (kg)	0.6850^b^	0.7155^a^	0.6800^c^	0.21
Feed conversion per egg dozen (kg)	1.4800	1.5700	1.4867	6.74
Laying hens viability (%)	100	100	100	12.40

The means in the lines followed by distinct lowercase letters indicate the difference between the diets test (p > 0.05). T1: Basal diet; T2: In the basal diet, 20% of yellow corn is replaced by wheat bran; T3: In the basal diet, 20% of yellow corn is replaced with wheat bran and soya bean oil. CV=Coefficient of variation

## Discussion

When compared to the control group (T1), replacing 20% of yellow maize with WB (T2) had no statistically significant differences (p > 0.05) in average live weight, egg laying rate, production of soft-shelled eggs, egg mass, feed conversion per dozen eggs, and laying hen viability. A different finding was described in a previous study [14] that looked at the performance effect of including up to 30% WB in the diet of laying hens. The difference between our findings and those of Araújo *et al*. [14] could be attributed to differences in laying hen age and WB-level incorporation. Furthermore, 20% WB inclusion did not appear to harm performance in laying hens, while 30% WB inclusion did.

When 20% of the yellow maize was replaced with WB, feed intake and feed conversion per egg mass increased (p < 0.05). The increased feed intake finding was unexpected because it was expected that the inclusion of WB, a high-fiber content ingredient, would limit feed intake due to low digestibility of the tract and thus low digesta passage rate, and thus increase the volume occupied in the digestive tract [15–17]. There are several explanations for this. With the addition of WB, the energy content of the feed decreased, and the laying hens may have consumed more to meet their energy requirements. Although the inclusion of WB in the diet increased the crude fiber content by one percentage unit (Table-1), the protein content increased by three percentage units; therefore, the protein may have overshadowed the fiber and there was no reduction in digestibility, increasing the passage rate and, as a result, an increase in feed intake [18].

The increase in protein content in experimental diets may have contributed significantly to the worsening of feed conversion in T2, as protein synthesis or nitrogen elimination in high-protein diets requires energy expenditure [19]. This implies that most of the energy in the diet was used for protein metabolism and only a small amount of energy was converted into egg mass. According to this argument, including soybean oil in T3 compensated for the energy deficit in the diet, resulting in improved feed conversion per egg mass (p < 0.05). The previous research by Sell *et al*. [20] and Brugalli *et al*. [21] supports this approach by demonstrating that adding lipids to laying hen diets improves feed energy efficiency, density, and palatability. These findings indicated that adding soybean oil improved feed efficiency and increased energy content, which was reduced when maize was replaced with solely WB.

## Conclusion

Adding 2.5% soybean oil to diets where WB replaces 17.5% yellow maize improved laying hen conversion rates. Therefore, the economic meaning of this outcome for laying hen feed costs is still to be addressed. Consequently, additional investigation program is required to evaluate the impact of adding soybean oil to diets where WB replaces maize in laying hen feed costs.

## Authors’ Contributions

MN, SCP, AT, EP, APC, LAJ, RT, FDA, and CGB: Conceptualization, validation, writing, review, and editing. MN, AT, AG, and MM: Assisted with animal care and data collection. MN, RT, and CGB: Methodology and statistical analysis. CGB: Project administration, resource allocation, funding, and supervision. All authors have read, reviewed, and approved the final manuscript.
